# Impact of Environmental Humidity on Instant Coffee Stability: Defining Moisture Thresholds for Quality Degradation and Shelf Life Prediction

**DOI:** 10.3390/foods14101826

**Published:** 2025-05-21

**Authors:** Marco Lopriore, Marilisa Alongi, Marika Valentino, Monica Anese, Maria Cristina Nicoli

**Affiliations:** Department of Agricultural, Food, Environmental, and Animal Sciences, University of Udine, Via Sondrio 2A, 33100 Udine, Italy; marco.lopriore@uniud.it (M.L.); marika.valentino@uniud.it (M.V.); monica.anese@uniud.it (M.A.); mariacristina.nicoli@uniud.it (M.C.N.)

**Keywords:** instant coffee, environmental relative humidity, T_g_, stability study, shelf life, quality decay, pH, solubility

## Abstract

Instant coffee powder is highly sensitive to environmental humidity, which can significantly affect its quality during storage. The objective of this study was to evaluate the storage stability of instant coffee by assessing the moisture uptake and monitoring the evolution of key quality indicators under different environmental relative humidity (ERH) conditions. To this purpose, instant coffee was removed from its original packaging and stored at 11, 32, and 65% relative humidity (ERH) at 20 °C. Quality parameters related to both the powder (visual appearance and solubilization time) and the resulting brew (pH) were monitored over time. The coffee stored at 11% ERH demonstrated stability throughout the observation period. Storage at 32% ERH resulted in short-term powder stability, but a notable pH decline after six months. At 65% ERH, critical moisture levels were exceeded within one week, resulting in rapid visual degradation, impaired solubilization, and reduced brew quality within three months. The acquired findings on the behavior of a dry food powder under various storage conditions are particularly relevant in the context of the increasing application of compostable packaging with low moisture barriers, in conjunction with the need to manage the secondary shelf life of dry food powders whose use is often parceled.

## 1. Introduction

The prevalence of coffee consumption around the world can be attributed to traditional consumption patterns and perceived physiological and supposed health benefits, which include stimulant properties, hypoglycemic effects, and antioxidant capacity [1]. Instant coffee represents the second most widely consumed coffee product on a global scale [2]. Coffee is produced by solid–liquid extraction of freshly ground-roasted coffee beans using hot water, followed by spray- or freeze-drying [3,4].

Instant coffee is a dry and highly hygroscopic powder, typically characterized by a moisture content of 2 to 4%. Consequently, the environmental relative humidity is the primary factor influencing the stability of instant coffee during storage and thus its shelf life [5]. When moisture exceeds certain thresholds, a series of physical and chemical events occur [6,7,8]. Caking is the predominant physical degradation effect triggered by moisture uptake [9], which causes a decrease in the coffee powder free flowing while reducing its water solubility. The critical moisture content at which caking occurs is around 5–7% [5]. In such conditions, storage temperature overcomes glass transition temperature thus generating a transition from a glassy to a rubbery amorphous state [10,11]. Such a transition causes an abrupt viscosity drop and a consequent increase of molecular mobility, possibly triggering a variety of chemical reactions, including the formation of caffeic acid upon chlorogenic acid hydrolysis, thus reducing the pH of the coffee brew. This results in a higher acidity perception, and compromises the acceptability of the brew [3,12,13,14,15,16,17].

These reactions are responsible for a decay in quality leading to an overall decrease in the acceptability of the coffee powder as well as the brew thereof obtained [18]. Although anti-caking agents, such as silicon dioxide and maltodextrin, can be used to improve powder flow properties and prevent caking during storage [19], avoiding moisture uptake remains crucial to guarantee the maintenance of an adequate quality level. Moreover, instant coffee must be packed using materials providing a high barrier against moisture [8]. Actually, instant coffee is commonly available on the market in formats fulfilling this requirement, such as in glass jars and in single dose multilayer flexible pouches (PET/Al foil/LDPE).

However, this scenario is expected to change in response to the European Parliament directives aimed at reducing single-use plastics to address environmental concerns about plastic waste [20,21]. Although the transition to sustainable alternatives is imperative, it concomitantly poses considerable challenges [22,23,24]. It has been demonstrated that even minor changes to the packaging solutions currently used for instant coffee can significantly accelerate a decay in the product quality. For instance, the elimination of the aluminium foil from the LDPE configuration resulted in a dramatic reduction of shelf life to 15 days [25], due to moisture uptake.

In the context of evolving packaging solutions, it is crucial to observe the behavior of the instant coffee powder when exposed to varying moisture conditions. Based on these considerations, the present study aimed to: (*i*) assess moisture uptake by the unpackaged instant coffee powder during storage under different environmental relative humidities (ERH); (*ii*) monitor the evolution of quality indicators of the instant coffee powder stored under ERH; (*iii*) measure the acidity of the brews obtained from the instant coffee powder stored under different ERH.

To this purpose, the instant coffee was stored at 11, 32, and 65% environmental relative humidity (ERH) at 20 °C, representative of some real-world scenarios (e.g., shipping, warehouse storage, kitchens), and some indicators accounting for the quality of the coffee powder, i.e., visual appearance and solubilization time, and of the brew, i.e., pH, were assessed.

## 2. Materials and Methods

### 2.1. Chemicals

Lithium chloride (LiCl, purity ≥ 99%) and magnesium nitrate hexahydrate (Mg(NO_3_)_2_ ∙ 6 H_2_O, purity ≥ 99%) were purchased from Merck KGaA (Darmstadt, Germany). Potassium acetate (C_2_H_3_O_2_K, purity = 98%), magnesium chloride hexahydrate (MgCl_2_ ∙ 6 H_2_O, purity ≥ 97%), potassium carbonate (K_2_CO_3_, purity ≥ 99%), and sodium nitrite (NaNO_2_, purity ≥ 97%) were purchased from Avantor (Radnor, PA, USA). Phosphorous pentoxide (P_2_O_5_, purity ≥ 100%) was purchased from Carlo Erba (Rodano (MI), Italy). Water was purified with a Milli-Q system (Millipore, Bedford, MA, USA).

### 2.2. Coffee Sample Preparation

Instant coffee (Nescafé Gold, Nestlé SA, Vevey, Switzerland) was purchased from a local supermarket. The product was packaged in transparent glass jars with a capacity of 100 g, representing the original commercial packaging. Instant coffee powder from the same production batch was removed from the original packaging and stored under different controlled environmental conditions. In particular, aliquots of 180 g of bulk instant coffee were introduced in aluminium trays (45 mm × 300 mm × 200 mm) and (30-µm thick) containers without lids. The sample was distributed to form a homogeneous layer of 5-mm thickness, and stored at 20 °C at three different environmental relative humidities, or ERH (%), i.e., 11, 32, and 65%. The ERH values were guaranteed by placing the samples on suspended perforated trays in hermetically closed plastic boxes containing a thin layer of supersaturated solutions of LiCl, MgCl_2_, and NaNO_2_. No direct contact between the saturated solution and the instant coffee occurred. The plastic boxes were then stored in dark conditions in a thermostatic incubator set at 20 ± 1 °C (Pol-Eko, Wodzislaw Slaski, Poland).

Samples were removed from the plastic boxes as time progressed during the storage and were monitored for moisture, color, solubility, and pH for the coffee brew obtained. The sampling frequency was defined on a case-by-case basis, depending on the results obtained at the previous sampling time, accordingly with the approach reported by other authors [18,26].

### 2.3. Moisture of Instant Coffee Powder

Moisture was determined by gravimetric method according to AOAC [27]. In particular, 1 g of the instant coffee powder was dried at 75 °C and 1.32 kPa for 12 h using a vacuum oven (Vuotomatic 50, Bicasa, Milan, Italy). After the drying period, the sample was cooled and weighed. The moisture content, or *M* (%), was calculated according to the following formula (Equation (1)):

(1) $$M\left(\%\right)=\frac{\left(w_{i}-w_{f}\right)}{w_{i}}\cdot 100$$

where $w_{i}$ is the initial weight of the sample (g) and $w_{f}$ is final weight of the sample (g) after the drying step. Data were reported as the average ± standard deviation of three measurements. The moisture uptake during storage was subsequently modeled using an empirical first order kinetic [28]:

(2) $$M\left(t\right)=M_{\infty }-({M_{\infty }-M}_{0})\cdot \exp\left(-k\cdot t\right)$$

where $M_{\infty }$ and $M_{0}$ represent the moisture content of the bulk instant coffee at time zero and at equilibrium, respectively, and *k* (day^−1^) is the moisture uptake rate of the instant coffee powder.

### 2.4. Water Activity (a_w_)

The instant coffee powder was measured using a dewpoint measuring instrument (AQUALAB 4TE, Astori Tecnica S.r.l., Poncarale, Italy). The results were expressed as the average ± standard deviation of three measurements.

### 2.5. Differential Scanning Calorimetry

Aliquots of 1 g of fresh instant coffee powder were put in small glass jars and placed on a suspended perforated tray in desiccators hermetically closed and containing supersaturated solutions of salts providing an ERH ranging from 5% to 65%. The salts used, with the corresponding ERH in brackets, were as follows: P_2_O_5_ (1%), LiCl (11%), CH_3_CO_2_K (23%), MgCl_2_ (32%), K_2_CO_3_ (43%), Mg(NO_3_)_2_ (54%), and NaNO_2_ (65%). Samples were stored at 20 °C until equilibrium with the ERH was reached.

Afterwards, amounts of 10 mg of sample were weighed in 100 µL aluminium pans. Aluminium pans were hermetically closed with aluminium lids and an empty aluminium closed pan was used as a reference. Samples were rapidly cooled down at −80 °C at 20 °C/min and heated from −80 to 160 °C using a scan rate of 10 °C/min.

Thermograms were analyzed for the onset temperatures of the glass transition (*T_g_*) using STAR^e^ software (v16.10). The results are reported as the average of at least 2 replicates, and the percentage of the coefficient of variation was less than 5%. The *T_g_* curve was fitted by the Gordon–Taylor parameter [29], according to Equation (3), as follows:

(3) $$T_{g}=\frac{kT_{gi}+\varepsilon WT_{gw}}{k+\varepsilon W}$$

where *T_gi_* and *T_gw_* are the glass transition temperatures of the anhydrous instant coffee powder and water, respectively, *k* and *W* are the mass fraction of the coffee solids (g_solids_/g_tot_) and water (g_water_/g_tot_), and ε is the Gordon–Taylor parameter. The model parameters *T_gi_* and ε were estimated while considering that the *T_gw_* is −135 °C.

### 2.6. Solubilization Kinetics of Instant Coffee Powder

The assessment of instant coffee powder solubility was performed by measuring the absorbance of the coffee beverage during the dissolution process. To this aim, 1.7 g of the coffee powder were solubilized in 100 mL of ultrapure water heated to 80 ± 2 °C using a thermostatic magnetic stirrer set at 20 rpm. Aliquots of 3 mL were withdrawn at 3, 10, 20, 30, 45, 60, and 120 s during the dissolution process, ensuring the exclusion of any undissolved powder or particulate matter. As no coffee powder residue was present in the collected samples, the dissolution process was effectively stopped at the moment of sampling. The aliquots were further diluted (1:100, *v*/*v*) with water for absorbance readings at 420 nm. The selected absorbance value was used as it represents a non-specific marker of Maillard reaction products, specifically high molecular weight compounds such as melanoidins. Absorbance was read at 20 °C using UV-Vis spectrophotometer (UV-2501 PC, Shimadzu, Kyoto, Japan), equipped with a 6-cell remotely controlled sample holder, thermoregulated by a Peltier system, using 1 cm path-length quartz cuvettes. The interval between sample collection and absorbance measurement was standardized (i.e., 1 min) to ensure consistency and comparability of the results. Data were expressed as the average ± standard deviation of at least 4 measurements.

Moreover, to estimate the apparent rate of instant coffee powder solubilization, the data were fitted using the following empirical model [28]:

(4) $$Abs\left(t\right)={Abs}_{\infty }-({{Abs}_{\infty }-Abs}_{0})\cdot \exp\left(-k\cdot t\right)$$

where ${Abs}_{\infty }$ and ${Abs}_{0}$ represent the absorbance value of the instant coffee brew at time zero and at equilibrium, respectively, and *k* (s^−1^) is the dissolution rate of the instant coffee powder in hot water.

### 2.7. pH of the Brew Obtained from Instant Coffee Powder

To determine the pH of the instant coffee beverage, 0.5 g of the instant coffee powder were solubilized with 25 mL of ultrapure water heated at 80 ± 2 °C. The solubilization was performed by means of a magnetic stirrer set at 20 rpm. After dilution, the coffee brew was rapidly cooled down to 20 °C by means of a blast chiller (Air-O-Chill, Electrolux Professional, Pordenone, Italy). The pH of the brew was measured with a pH-meter (Basic20, Crison Instruments, Barcelona, Spain). The results are reported as the average ± standard deviation of 3 measurements.

To model the evolution of the pH decay rate, the experimental observations were modeled by using a pseudo-first order kinetics as follows:

(5) $$pH\left(t\right)={pH}_{0}\cdot \exp\left(-k\cdot t\right)$$

where ${pH}_{0\;}$ is the value of the pH at time zero and *k* (day^−1^) is the value of the constant rate of pH decay during storage.

### 2.8. Adsorption Isotherm of Instant Coffee Powder

The adsorption isotherm was carried out utilizing ProUmid “Vsorp Basic” dynamic vapor sorption analyzer system (ProUmid GmbH & Co. KG, Ulm, Germany). An aliquot of 4 ± 1 g of the instant coffee powder was placed on an aluminium plate and placed within the climatic chamber of the instrument at 20 ± 1 °C. The *a_w_* in the sample headspace was automatically increased from 0 to 0.9 ± 0.01, with 0.1 *a_w_* steps. The device performed automatically the measure of the samples’ weight every 20 min with a reproducibility of 0.1 mg. The equilibrium of each step was reached when the sample mass variation was lower than 0.01% for 8.5 h. The moisture content was expressed as g_water_/g_db_, in accordance with standard practice to enable meaningful mathematical modeling [30,31]. Equilibrium moisture data were finally modeled by using the Guggenheim–Anderson–De Boer (GAB) equation (Equation (6)) as follows:

(6) $$M=\frac{m_{0}\cdot b\cdot C\cdot a_{w}}{\left(1-b\cdot a_{w})\cdot (1-b\cdot a_{w}+C\cdot b\cdot a_{w}\right)}$$

where *M* is the moisture content expressed as the ratio of the mass of water per mass of dry matter (g_water_/g_db_), *m*_0_ represents the moisture content at monolayer level (g_water_/g_db_), and *b* and *C* are fitting parameters.

### 2.9. Color

A color analysis was carried out on the instant coffee powder using a tristimulus colorimeter (Chromameter-2 Reflectance, Minolta, Osaka, Japan) equipped with a CR-300 measuring head. The instrument was standardized against a white tile before measurement. Therefore, 6.5 g of the instant coffee powder were milled, placed in a Petri dish, and the color was measured as the average ± standard deviation of 5 measurements. The color was finally expressed in CIE units as L* (lightness/darkness), a* (redness/greenness), and b* (yellowness/blueness).

### 2.10. Image Acquisition and Analysis

Images of the instant coffee powder were taken using a Canon EOS 550D digital reflex camera (Canon Inc., Tokyo, Japan) mounted on an adjustable stand positioned 50 cm above the Petri dish containing the sample. An image analysis was performed using Image-Pro Plus software (ver. 6.3, Media Cybernetics, Inc., Bethesda, Rockville, MD, USA) to determine a freshness index for the coffee powder, based on a previously applied approach with some modifications [32]. The RGB intervals describing the fresh samples were determined by screening an image of a fresh sample and selecting the relevant RGB ranges [26]. For the instant coffee powder, these ranges corresponded to R (70–190), G (55–170), and B (10–90).

These intervals were used to convert each image into a binary mask in which all pixels with colorimetric parameters within the selected ranges appeared white, indicating a ‘fresh’ appearance, while those outside the ranges appeared black. The white and black pixels were then quantified using the software. The freshness index was calculated by determining the percentage ratio between the number of white pixels at each storage time and the number of white pixels at time zero, with the value at time zero normalized to represent 100% freshness.

### 2.11. Statistical Analysis

The results are reported as the mean ± standard deviation, calculated from three replicated samples. Data were analyzed using two distinct analysis of variance (ANOVA) approaches. To evaluate differences among the samples over increasing storage times, a one-way repeated measures ANOVA followed by Tukey’s Honest Significant Differences test (*p* < 0.05) was applied. For comparisons between samples stored under different ERH conditions but analyzed after the same storage period, a one-way between-subjects ANOVA with the Tukey HSD test (*p* < 0.05) was conducted. The nonlinear fitting of data was performed by using the library nlstools (version 2.1-0) [33]. All the statistical computations were conducted using R version 4.3.2 for Windows (The R foundation for statistical computing, Vienna, Austria) through the RStudio environment (version 2023.09.1-494).

## 3. Results and Discussion

### 3.1. Moisture Uptake by Instant Coffee Powder During Storage Under Different Environmental Humidities

Instant powders, such as instant coffee, are highly porous products that exhibit a strong ability to be rehydrated. This characteristic is compromised when the moisture content of the matrix exceeds a certain threshold. To investigate the rate and the extent of moisture uptake, the moisture content of bulk instant coffee powder was monitored during storage at different ERH values.

A broad range of the ERH, representative of the conditions reasonably experienced by the instant coffee during shipping, distribution, and domestic storage (i.e., 11, 32, and 65% at 20 °C) was considered. The worst-case scenario condition, i.e., 65% ERH, is commonly encountered in domestic settings or in storage facilities where no controlled environmental conditions are put in place.

**Figure 1:**
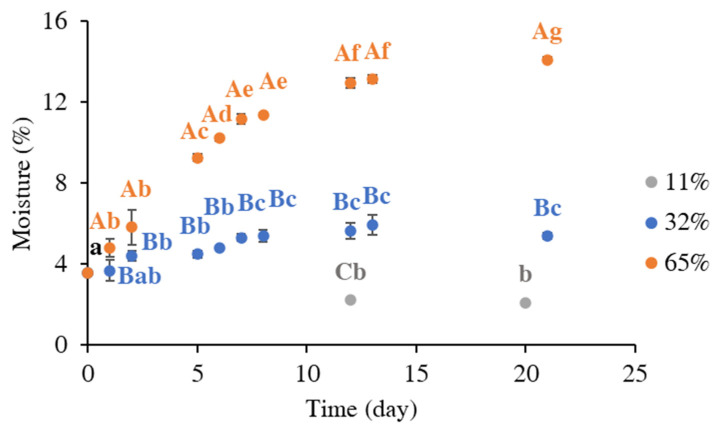
Moisture (%) uptake of the instant coffee powder during storage at 11, 32, and 65% ERH at 20 °C. Uppercase letters denote significant differences (*p* < 0.05) between the samples stored at varying ERH levels within the same storage time (one-way ANOVA). Lowercase letters reflect significant temporal differences (*p* < 0.05) in the moisture content at each ERH condition (repeated measures ANOVA).

Figure 1 shows the experimental data relevant to the moisture (%) uptake of the instant coffee powder during storage at 11, 32, and 65% ERH at 20 °C, and Table 1 reports the corresponding parameter estimates obtained by first-order kinetics modeling.

The results reported in Figure 1 display the progressive increase of moisture (%), or *M*, of the instant coffee powder.

The *M* value of the fresh instant coffee was 3.58 ± 0.02%, corresponding to water activity (*a_w_*) of 0.13 ± 0.05%. As expected, the extent of the moisture uptake upon different storage conditions depended on the ERH values. When the instant coffee was stored at 11% ERH, representing a low environmental humidity condition, the moisture content remained constant during the whole storage timeframe, with an *M* value of 2.10 ± 0.05% after 19 days of storage (Figure 1). This result was confirmed by the low capacity of the model to fit experimental data, as demonstrated by the RMSE relevant to the sample stored at 11% ERH (Table 1). Samples stored at 32% ERH reached equilibrium with a moisture content of 5.41 ± 0.13% within 8 days, while those stored at 65% ERH reached equilibrium at 14.11 ± 0.11% of moisture within 20 days. It can be noticed that the rate of moisture uptake was comparable between the samples stored at 32 and 65% ERH (Table 1). In fact, it can be noticed that the sample stored at 32% ERH reached its plateau much earlier compared to the sample stored at 65% ERH (Figure 1).

**Figure 2:**
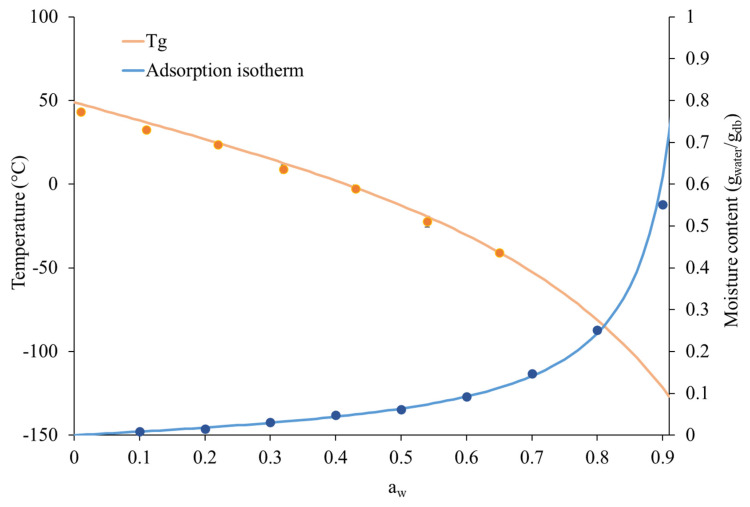
Modified state diagram of the instant coffee powder showing the glass transition temperature curve (*T_g_*) and the adsorption isotherm at 20 °C as a function of water activity (*a_w_*). Continuous orange and blue lines represent the best fitting of Equations (3) and (6) on glass transition temperature (burnt orange dots) and equilibrium moisture content (dark blue dots) data, respectively.

The recorded moisture uptake can lead to considerable changes in the physical state of a dry food product, such as instant coffee powder [10,18]. To depict the physical state of the product when stored under different ERH values, the modified state diagram of the instant coffee powder was determined. The obtained modified state diagram is shown in Figure 2, where the onset glass transition temperature is plotted as the function of *a_w_*, together with its adsorption isotherm.

The slope of the adsorption isotherm curve progressively increased as soon as the water activity increased. The GAB constants *C* and *b* were found to be equal to 1.10 and 0.99, respectively, and the moisture content at monolayer level (*m_0_*) was 0.06 g_water_/g_db_, corresponding to an *a_w_* of 0.48 at the monolayer (*a_w0_*) and a moisture percentage of 6.22%. The obtained results well aligned with previous findings reported in the literature [10,34]. Based on this evidence, and considering the evolution of moisture uptake under different environmental humidities (Figure 1), it can be observed that only samples stored at 65% ERH were able to reach and overcome the moisture percentage corresponding to the monolayer within approximately five days.

The modified state diagram (Figure 2) also shows the *T_g_* of the coffee samples as a function of *a_w_* that is related to different ERH storage conditions. As expected, the glass transition temperature of the instant coffee powder decreased as the *a_w_* increased. Experimental *T_g_* data were fitted according to the Gordon–Taylor model (Equation (3)). The parameters relevant to the anhydrous instant coffee powder, i.e., *T_gi_* and *k*, were 47.8 °C and 7.2, respectively. When the instant coffee reached the monolayer, the *T_g_* decreased significantly to a value of −9.1 °C.

Under the storage conditions examined in this study, instant coffee stored at 32% ERH would be expected to have a glass transition temperature (*T_g_*) of 14 °C, which is close to the storage temperature recommended for this product, and is the value considered in the present study (i.e., 20 °C). Comparable results on the soluble coffee powder were previously reported by Anese and co-workers [10], where the sample equilibrated at 35% ERH showed a *T_g_* equal to 25 °C. In this regard, it must be pointed out that a ΔT (i.e., *T* − *T_g_*) less than or equal to 5 °C is not sufficient to account for a complete phase transition from glass to rubber [35]. Conversely, if the coffee powder was stored at 65% ERH, a *T_g_* of −41 °C would be expected. This means that, at a typical room temperature, which is the recommended storage temperature for this product, the instant coffee powder stored at 65% ERH would be in a rubber state. This condition could easily be achieved in domestic settings (e.g., kitchens and cellars). Even at low *a_w_* values (i.e., 0.1), exposure to extreme environmental temperatures (e.g., 45 °C) can induce a glass-to-rubber transition (ΔT = 10 °C). This scenario typically occurs during shipping and warehouse storage in hot climates [36].

### 3.2. Evolution of Quality Indicators of Instant Coffee Powder Stored Under Different Environmental Humidities

The glass–rubber transition suffered by the instant coffee powder when stored at 32% and 65% ERH is supposed to affect its technological performance and consumer experience. Based on this consideration, we investigated the evolution of some quality attributes of the instant coffee powder, which are expected to be influenced by its physical state. Specifically, we considered the visual appearance that is known to be affected by moisture uptake [37], and the dissolution time in hot water, representative of the rehydration performance, and thus the ease of use [38].

#### 3.2.1. Effect on the Visual Appearance

No remarkable changes were observed in the visual appearance of the instant coffee stored at 11 and 32% ERH, whereas storage at 65% ERH led to a progressive decrease in the brightness of the powder (Figure S1).

In this regard, the glass–rubber transition occurring at 65% ERH (Figure 2) leads to the formation of liquid bridges between the particles, increasing cohesiveness [39,40,41]. This in turn affects the optical characteristics of the powder and, specifically, its light-scattering properties, contributing to the observed decrease in the brightness [39] associated with coffee staling [42].

**Figure 3:**
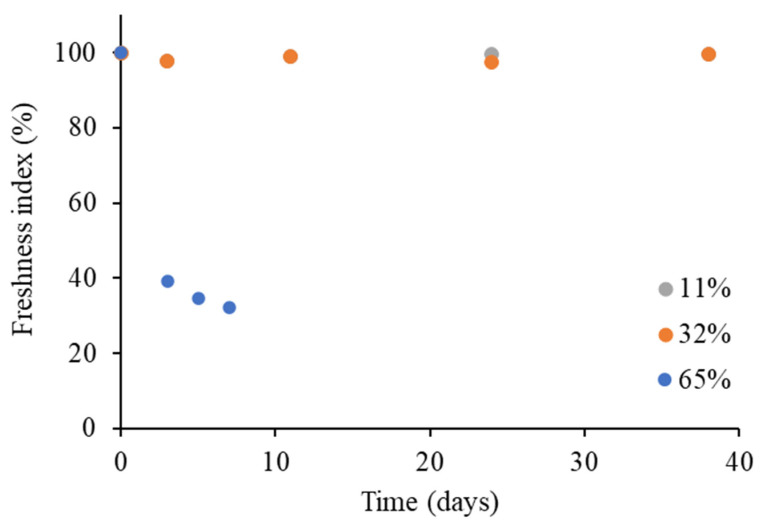
Freshness index of the instant coffee powder during storage at 11, 32, and 65% ERH at 20 °C.

In order to quantify the visually observed changes, the coffee powder images were processed by image analysis to obtain a “freshness indicator” (Figure 3).

The freshness index, corresponding to 100% in the coffee powder at the beginning of storage, demonstrated a steady progression for samples stored at 11 and 32% ERH, whereas this index experienced a sharp decline when the instant coffee was stored at 65% ERH, reaching 31% within just one week. This result underscores that the storage of the instant coffee under conditions exceeding *a_w_*_0_ and *T_g_* (Figure 2) exerts a marked influence on its visual characteristics, ultimately compromising consumer acceptance. It is also worth noting that the latter scenario (i.e., 20 °C and 65% ERH) closely reflects the average conditions found in a typical domestic kitchen environment. The evolution of the freshness index discussed here highlights the susceptibility of the instant coffee to quality deterioration under common household and foodservice storage conditions. This is particularly relevant in the context of the product’s secondary shelf life, during which packages are frequently opened and closed, and typically consumed in a timespan longer than one week [30].

**Figure 4:**
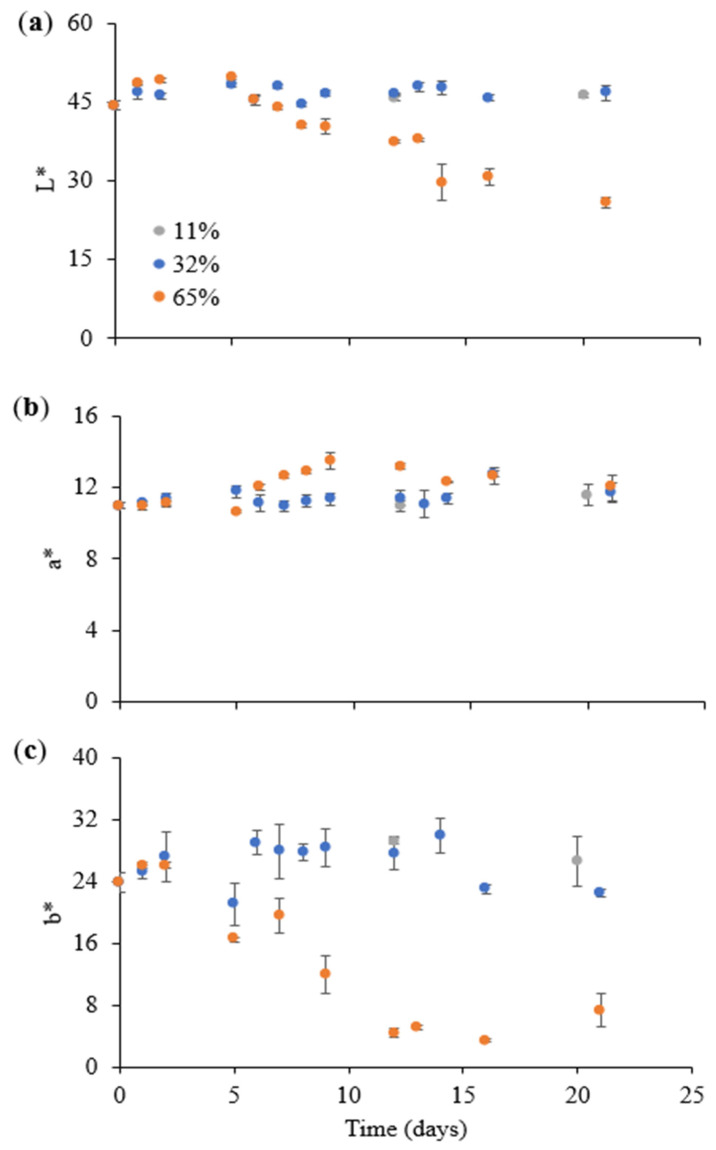
CIE color parameters, i.e., L* lightness (**a**), a* red–green (**b**), and b* yellow–blue (**c**) of the instant coffee powder during storage at 11, 32, and 65% ERH at 20 °C.

To further illustrate the changes in the appearance of the instant coffee exposed to varying ERH values, the color evolution (L*, a*, b*) of the instant coffee powder during storage at 11, 32, and 65% ERH at 20 °C is displayed in Figure 4, and significant differences among sample are listed in Table S1.

Considering samples stored at 11 and 32% ERH, the color parameters remained almost unchanged during the whole storage timespan (Figure 4), in line with the freshness index (Figure 3), confirming the stability of the product stored under these conditions. Conversely, in the case of the sample stored at 65% ERH, a significant decrease in the instant coffee luminosity (L*) and yellowness (b*) was observed within the first 15 days, and was followed by a plateau for both parameters (Table S1).

As previously discussed, the progressive decline in L* and b* values can be reasonably attributed to the glass–rubber transition, which alters the optical properties due to moisture uptake [39,40,41]. The correlation between moisture content and both color parameters was assessed, revealing a strong negative correlation between moisture and L* (*r* = 0.73), as well as between moisture and b* (*r* = 0.85). Notably, the observed color changes (Figure 4) occurred at a slower rate than the decline in the freshness index (Figure 3). Accordingly, a strong negative correlation (*r* = 0.81) was also found between the moisture content and the freshness index, suggesting that the latter may serve as a rapid and reliable indicator of moisture uptake in the instant coffee powder.

#### 3.2.2. Effect on Rehydration Performance

**Figure 5:**
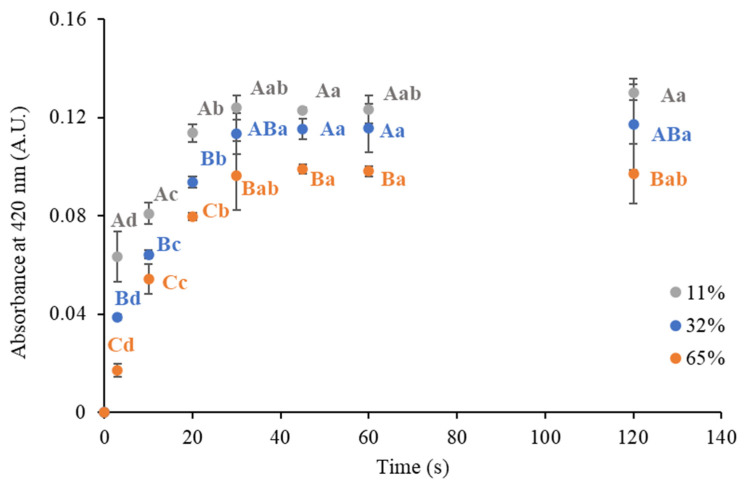
Absorbance at 420 nm at different solubilization times of the brew obtained from the instant coffee powder equilibrated at 11, 32, and 65% ERH at 20 °C. Uppercase letters identify significant differences (*p* < 0.05) between the samples stored at different ERH levels at the same solubilization time, as determined by one-way ANOVA. Lowercase letters indicate significant differences (*p* < 0.05) across the solubilization times for each ERH condition, analyzed using repeated measures ANOVA.

The rehydration performance of samples stored at 11, 32, and 65% ERH was assessed. In particular, the solubilization of the coffee soluble compounds was monitored by measuring the absorbance at 420 nm during different times. Figure 5 shows the relevant experimental data, and Table 2 reports the corresponding parameter estimates obtained by empirical modeling.

Considering the model estimates, it can be noticed that the sample stored at 11% ERH presented a higher apparent rate of solubilization compared to those stored at 32 and 65% ERH (Table 2). This effect can be attributed to changes in the physical state (Figure 1), such as stickiness and structural collapse, which compromise the initial rehydration performance of the instant coffee powder.

After 2 min of stirring the rehydration performance of the instant coffee powder was only slightly affected by the ERH, except for the worst-case scenario (i.e., 65% ERH), which showed a significant difference with the sample equilibrated at 11% ERH. Nevertheless, it should be considered that the typical stirring time adopted by consumers when preparing hot beverages from instant powders hardly exceeds 30 s [43]. After this time, the solubilization of the samples equilibrated at 65% ERH seemed to be hindered. This effect can be attributed to changes in the physical state (Figure 1), such as stickiness and structural collapse, which compromise the initial rehydration performance of the instant coffee powder.

The significant difference among the samples provided evidence that the user experience during solubilization may be affected by the storage conditions to which the bulk instant coffee is exposed. Also, in this case, sensory tests should be carried out to verify the actual impact on the consumers’ acceptability.

### 3.3. Changes in the pH of Brews Obtained from Instant Coffee Powder Stored Under Different Environmental Humidities

In addition to directly affecting the performances of the coffee powder, moisture uptake has been shown to trigger hydrolytic events leading to a decrease in the pH of the brew resulting in a reduced sensory acceptability [13,44,45,46,47].

**Figure 6:**
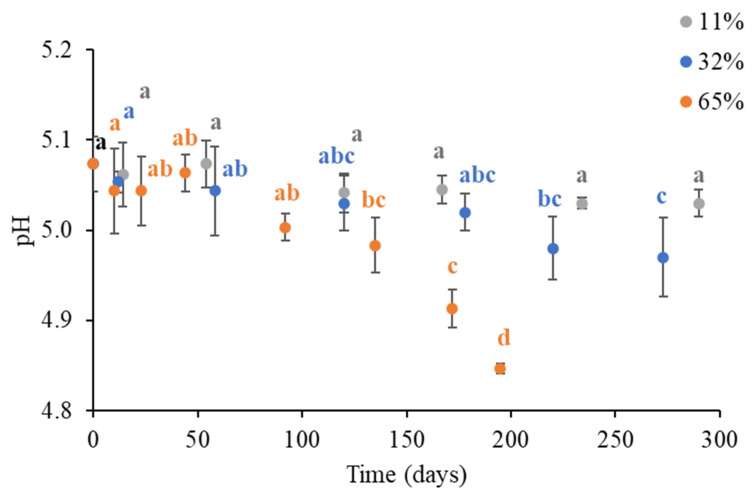
The pH of the coffee brew obtained from the instant coffee powder during storage at 11, 32, and 65% ERH at 20 °C. Lowercase letters (a–d) indicate temporal significant differences (*p* < 0.05) in the pH means at each ERH condition, analyzed using repeated measures ANOVA.

The evolution of the pH of the coffee brew obtained from the instant coffee powder stored for increasing time at 11, 32, and 65% ERH at 20 °C was thus monitored, and the experimental results and the corresponding parameter estimates obtained by empirical modeling are reported in Figure 6 and Table 3, respectively.

The pH of the brew obtained from the fresh instant coffee powder was 5.07 ± 0.03. This value is lower than the pH commonly measured in the fresh coffee brews (from 5.2 to 5.5), and is close to the acceptability limit attributed to the coffee brew, namely, 5.1 [18,45,46].

Nevertheless, it should be pointed out that the pH of the brew obtained from instant coffee is typically lower compared to the brewed coffee made from fresh grounds due to changes in the organic acid composition during processing. While acids, such as acetic, citric, formic, malic, and chlorogenic, decrease in concentration, acids, such as quinic acid, phosphoric acid, and pyroglutamic acid, increase [48]. In particular, phosphoric acid exerts a significant influence on acidity due to its high pKa, thereby contributing to the higher total acidity percentage observed in the soluble coffee [49].

The pH remained unaffected when the instant coffee was exposed to recommended “dry” storage conditions at 11% ERH, while storing the product at a higher humidity caused a decrease in the pH of the brew (Figure 6). The rate of pH decrease suffered almost a 3-fold and a 5-fold increase when the ERH passed from 11% to 32 and 65%, respectively, confirming the critical effect of moisture uptake in compromising the quality of the coffee. The pH decrease was noticeable (pH < 5) after 180 days of storage already at 32% ERH, and occurred faster at 65% ERH, with a pH < 5 around 135 days (Table S2).

This behavior perfectly aligns with the existing literature, where a strong correlation between the pH of the coffee extract and the moisture content of the coffee powder has been reported [18,45,46]. Such a pH decay was attributed to the evolution of the Maillard reaction in products to acidic compounds that are favored when environmental conditions (i.e., ERH and temperature) exceed the monolayer and *T_g_* (Figure 1) [13,18,46].

## 4. Conclusions

This study demonstrated that environmental humidity plays a crucial role in determining the stability and quality of the instant coffee powder during storage. Coffee stored at 11% ERH remained stable, while at 32% ERH, short-term stability was maintained, but a significant pH reduction occurred after six months. At 65% ERH, the coffee powder exceeded critical moisture levels (*m*_0_) within one week, leading to rapid visual deterioration, compromised solubilization performance, and a decline in the brew quality within three months. These findings underline the importance of storage conditions in maintaining the coffee quality, particularly in real-life settings (e.g., shipping, warehousing, kitchens) where the product may be exposed to different environmental conditions and its consumption is reasonably parceled, which raises the issue of secondary shelf-life. Beyond the observed results, this study suggests that exceeding the *m*_0_ accelerates quality degradation more rapidly than temperature-induced glass–rubber transitions. Furthermore, the findings suggest that the deterioration in solubilization performance and visual appearance precedes the reduction in pH, and may serve as indicators of early-stage quality deterioration. It is noteworthy that, even under moderately stable conditions (32% ERH), subtle quality changes can occur that are only discernible through sensory evaluation.

The findings of this study have the potential to inform the definition of the shelf life of the instant coffee in relation to storage conditions. In this context, the collected data offer valuable insights into the selection of optimal packaging materials and storage conditions to ensure quality retention over time.

To ensure the accuracy of shelf-life determination, future research should focus on precisely establishing acceptability thresholds for pH, solubility, and the coffee powder appearance. Furthermore, the potential of imaging techniques as a practical alternative to traditional moisture measurement methods should be explored, with a view to developing a rapid and efficient method for monitoring bulk powder quality during storage.

## Figures and Tables

**Table 1:** Estimated initial moisture value ($M_{0}$), equilibrium moisture value ($M_{\infty }$), and apparent constant rate of the instant coffee moisture uptake (k) at increasing environmental relative humidity levels (ERH%) of the bulk instant coffee moisture uptake. The goodness of fitting is expressed using the root mean square error (RMSE).

ERH (%)	$M_{0}$	$M_{\infty }$	*k* (Day^−1^)	RMSE
11	3.5 ± 0.1	2.8 ± 0.5	0.08 ± 0.12	1.21
32	3.5 ± 0.2	5.8 ± 0.2	0.18 ± 0.06	0.24
65	3.3 ± 0.2	14.7 ± 0.3	0.15 ± 0.01	0.22

**Table 2:** Estimated initial absorbance value (${Abs}_{0}$), equilibrium absorbance value (${Abs}_{\infty }$), and apparent constant rate of the instant coffee solubilization (k) by means of fitting experimental observations at different environmental relative humidity levels (ERH%) using Equation (4). The goodness of fitting was assessed using the root mean square error (RMSE).

ERH (%)	${Abs}_{0}$	${Abs}_{\infty }$	*k* (s^−1^)	RMSE
11	0.008 ± 0.009	0.124 ± 0.005	0.13 ± 0.03	0.008
32	0.005 ± 0.005	0.118 ± 0.003	0.08 ± 0.01	0.004
65	0.002 ± 0.002	0.101 ± 0.002	0.07 ± 0.01	0.003

**Table 3:** Estimated initial pH value (${pH}_{0}$) and constant rate of pH decay (k) using fitting experimental observations at different environmental relative humidity (ERH%) using a pseudo-first order kinetic model. The goodness of fitting was assessed using the root mean square error (RMSE).

ERH (%)	${pH}_{0}$	k	RMSE
11	5.07 ± 0.01	0.38 ± 0.97	0.021
32	5.07 ± 0.01	0.72 ± 0.11	0.024
65	5.08 ± 0.01	2.11 ± 0.21	0.035

## Data Availability

The original contributions presented in the study are included in the article/Supplementary Material, further inquiries can be directed to the corresponding author.

## Supplementary Materials

The following supporting information can be downloaded at: https://www.mdpi.com/article/10.3390/foods14101826/s1, Figure S1: Changes in visual appearance of instant coffee during storage at 20 °C at different environmental relative humidity levels (ERH); Table S1: CIE colour parameters; Table S2: pH of the coffee brew obtained from instant coffee powder during storage at 11, 32 and 65% ERH at 20 °C.
