# Supplementary material for: Impact of Environmental Humidity on Instant Coffee Stability: Defining Moisture Thresholds for Quality Degradation and Shelf Life Prediction
Source: Foods. 2025 May 21;14(10):1826. doi: 10.3390/foods14101826 (PMC12111376; doi:10.3390/foods14101826)
Supplement: Supplementary file 1 [file foods-14-01826-s001.zip › foods-3592428-supplementary.pdf]

Time (days) / ERH (%)	0	3	38
11	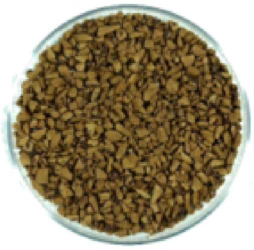	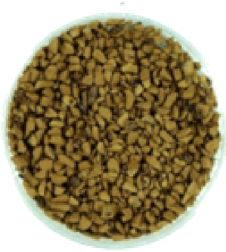	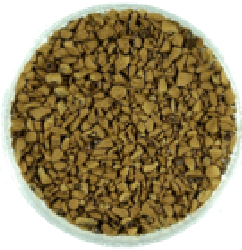
32	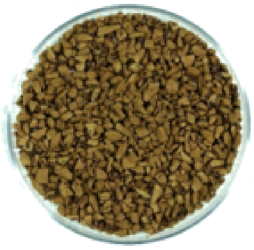	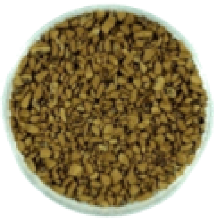	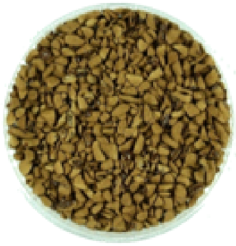
65	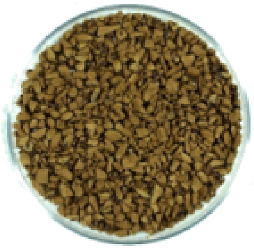	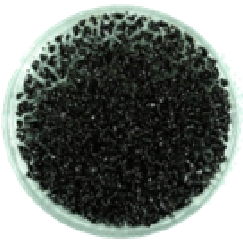	**N.A.**

Figure S1. Changes in visual appearance of instant coffee during storage at 20 °C at different environmental relative humidity levels (ERH).

Table S1. CIE colour parameters, *i.e.* L* lightness (a), a* red-green (b), and b* yellow-blue (c) of instant coffee powder during storage at 11, 32 and 65% ERH at 20 °C.

ERH (%)	Time	L*	a*	b*
11	0	44.4 ± 0.9^{Ab}	11.0 ± 0.2^{Aa}	23.9 ± 1.3^{Ab}
	12	45.8 ± 0.6^{Aab}	11.0 ± 0.3^{Aa}	29.2 ± 0.4^{Aa}
	20	46.3 ± 0.5^{Aa}	11.5 ± 0.6^{Aa}	26.6 ± 3.2^{Aab}
32	0	44.4 ± 0.9^{Ad}	11.0 ± 0.2^{Aa}	23.9 ± 1.3^{Aabcd}
	1	46.8 ± 1.3^{Aabc}	11.1 ± 0.1^{Ab}	25.2 ± 09^{Aabcd}
	2	46.1 ± 0.6^{Abcd}	11.3 ± 0.4^{Ab}	27.2 ± 3.2^{Aabc}
	5	48.2 ± 0.4^{Aa}	11.7 ± 0.3^{Ab}	21.1 ± 2.7^{Ad}
	6	45.3 ± 1.0^{Acd}	11.1 ± 0.4^{Ab}	29.0 ± 1.6^{Aab}
	7	47.9 ± 0.4^{Aab}	10.9 ± 0.3^{Ab}	27.9 ± 5.5^{Aabc}
	8	44.4 ± 0.4^{Ad}	11.2 ± 0.3^{Ab}	27.7 ± 1.0^{abc}
	9	46.6 ± 0.6^{Aabc}	11.3 ± 0.4^{Ab}	28.4 ± 2.5^{Aabc}
	12	46.5 ± 0.3^{Aabc}	11.4 ± 0.4^{Bb}	27.7 ± 2.1^{Aabc}
	13	47.9 ± 0.9^{Aab}	11.1 ± 0.7^{b}	N.A.
	14	47.7 ± 1.3^{Aab}	11.4 ± 0.3^{Ab}	29.9 ± 0.5^{a}
	16	45.7 ± 0.6^{Acd}	12.8 ± 0.2^{Ab}	22.9 ± 0.4^{Abcd}
	20	46.8 ± 1.4^{Aabc}	11.7 ± 0.5^{Ab}	22.9 ± 3.7^{Ad}
65	0	44.4 ± 0.9^{Ab}	11.0 ± 0.2^{Ae}	23.9 ± 1.3^{Aab}
	1	48.7 ± 0.3^{Aa}	10.9 ± 0.1^{Be}	26.2 ± 0.3^{Ba}
	2	49.1 ± 0.4^{Ba}	11.1 ± 0.2^{Ae}	26.1 ± 0.4^{Aa}
	5	49.6 ± 0.4^{Ba}	10.6 ± 0.1^{Be}	16.5 ± 0.3^{Acd}
	6	45.2 ± 0.8^{Ab}	12.0 ± 0.1^{Bd}	24.4 ± 2.2^{Ba}
	7	43.9 ± 0.5^{Bb}	12.6 ± 0.1^{Bbcd}	19.5 ± 2.5^{Bbc}

	8	40.5 ± 0.5^{Bc}	12.9 ± 0.1^{Babc}	N.A.
	9	40.3 ± 1.3^{Bc}	13.5 ± 0.5^{Ba}	12.0 ± 0.3^{Bd}
	12	37.5 ± 0.2^{Bd}	13.2 ± 0.2^{Bab}	4.5 ± 0.2^{Ae}
	13	37.8 ± 0.3^{Bcd}	N.A.	5.0 ± 2.1^{e}
	14	29.7 ± 3.4^{Be}	12.3 ± 0.4^{Bcd}	N.A.
	16	30.7 ± 1.6^{Be}	12.6 ± 0.7^{Abcd}	3.5 ± 1.1^{Be}
	20	25.8 ± 0.9^{Bf}	12.0 ± 0.8^{Ad}	N.A.

$^{a\text{-}f}$ For the same CIE colour parameter means within the same ERH (*i.e.*, at increasing stoarage times) indicated by different letters are significantly different ($p < 0.05$). $^{A\text{-}C}$ For the same CIE colour parameter means within the same storage time at different ERH indicated by different letters are significantly different ($p < 0.05$)

Table S2. pH of the coffee brew obtained from instant coffee powder during storage at 11, 32 and 65% ERH at 20 °C. $^{a-c}$ For the pH means within the same ERH (*i.e.*, at increasing stoarage times) indicated by different letters are significantly different ($p < 0.05$).

ERH (%)	Time	pH
11	0	5.07 ± 0.03^{a}
	14	5.06 ± 0.04^{a}
	54	5.07 ± 0.03^{a}
	120	5.04 ± 0.02^{a}
	167	5.05 ± 0.02^{a}
	234	5.03 ± 0.01^{a}
	290	5.03 ± 0.02^{a}
32	0	5.07 ± 0.03^{a}
	12	5.05 ± 0.01^{a}
	58	5.04 ± 0.05^{ab}
	120	5.03 ± 0.03^{abc}
	178	5.02 ± 0.02^{abc}
	220	4.98 ± 0.03^{bc}
	273	4.97 ± 0.04^{c}
65	0	5.07 ± 0.03^{a}
	10	5.04 ± 0.05^{a}
	23	5.04 ± 0.04^{ab}
	44	5.06 ± 0.02^{ab}
	92	5.00 ± 0.02^{ab}
	135	4.98 ± 0.03^{bc}
	172	4.91 ± 0.02^{cd}
	195	4.85 ± 0.01^{d}
